# Literary Notes

**Published:** 1903-04

**Authors:** 


### LITERARY NOTES.

*Dental Materia Medica, Therapeutics and Prescription Writing* is the title of a treatise of which Professor Eli H. Long, M. D., of Buffalo, is the author. It is a standard octavo volume of 333 pages and is intended as a guide for dental students and practitioners in the topics named in its title. The book has been issued recently from the press of Lea Brothers, Philadelphia, and is in the good style and form for which that house is celebrated. Price, cloth, $3.00.

---

The Standard Medical Directory for 1903, is now going through the press. It is a high class book of reference, judged by the volume of last year, and it is anticipated the current volume will far excel the previous issue. It will consist of about 13,00 pages comprising complete directories respectively of the physicians of all North America, colleges, societies, hospitals, sanitariums, mineral springs, publications and in fact everything related to medicine. The new features including an alphabetical index of physicians with post-office addresses and rosters of practitioners of the specialties will, it is stated, add about one-third to the volume of the work. Published by G. P. Engelhard & Co., Chicago.

THE *Bulletin of the Free Hospital for Women*, is the organ of a charity located at Boston, Mass., which presented itself in March. Its first number contains material of gynecological interest. The Bulletin is under the immediate supervision of Dr. William P. Graves, who is announced as assistant editor and resident pathologist at the hospital. Dr. Edward Reynolds is visiting surgeon and editor.

THE eleventh annual report of the Charity Eye, Ear, and Throat Hospital, for the year ending September 30, 1902, presents an interesting exhibition of efficient charity work by the physicians attached to the service staff. It appears that during the period covered by the report, 1,337 eye cases, 278 ear cases, and 157 nose and throat cases, besides a number of miscellaneous cases, have been treated.

